# Clinical Practice Recommendations on Kidney Management in Methylmalonic Acidemia: an Expert Consensus Statement From ERKNet and MetabERN

**DOI:** 10.1016/j.ekir.2024.09.002

**Published:** 2024-09-06

**Authors:** Aude Servais, Miriam Zacchia, Laurène Dehoux, Rukshana Shroff, Anais Brassier, Roberta Taurisano, Stefan Kölker, Jun Oh, Gema Ariceta, Jelena Stojanovic, Friederike Hörster, Dello Strologo, Marco Spada, Manuel Schiff, Carlo Dionisi-Vici

**Affiliations:** 1 Nephrology and Transplantation Department, Inherited Kidney Diseases Reference Center, Necker-Enfants Malades University Hospital, Assistance Publique Hôpitaux de Paris, Inserm U1163, Imagine Institute, Université de Paris, Paris, France; 2 Department of Medical and Translational Sciences, University of Campania, Luigi Vanvitelli, Naples, Italy; 3 Pediatric Nephrology Department, Necker-Enfants Malades University Hospital, Assistance Publique Hôpitaux de Paris, Paris, France; 4 Institute of Child Health University College London, Great Ormond Street Hospital, NHS Foundation Trust, London, UK; 5 Inherited Metabolic Diseases Reference Center, Necker-Enfants Malades University Hospital, Assistance Publique-Hôpitaux de Paris, Université Paris Cité, Paris, France; 6 Division of Metabolic Diseases and Hepatology, Bambino Gesù Children's Hospital, IRCCS, Rome, Italy; 7 Division of Pediatric Neurology and Metabolic Medicine, Center for Pediatric and Adolescent Medicine, Heidelberg University, Medical Faculty, European Network for Hereditary Metabolic Disorders, Heidelberg, Germany; 8 University Medical Center Hamburg-Eppendorf, Hamburg, Germany; 9 Department of Pediatric Nephrology, Hospital Vall d'Hebron, Universitat Autonoma Barcelona, Barcelona, Spain; 10 Nephrology department, Bambino Gesù Children's Hospital IRCCS, Rome, Italy; 11 Division of Hepatobiliopancreatic Surgery, Liver and Kidney Transplantation; Bambino Gesù Children's Hospital, IRCCS, Rome, Italy

**Keywords:** combined liver-kidney transplantation, daily dialysis, methylmalonic acidemia, neurotoxicity

## Abstract

Methylmalonic acidemias (MMAs) are rare inherited metabolic diseases with multiorgan involvement. Chronic kidney disease (CKD) is a common complication, leading to kidney failure, dialysis, and kidney transplantation (KT). The objective of these guidelines was to develop clinical practice recommendations focusing on specific aspects of the kidney management of this disease.

Development of these clinical practice recommendations is an initiative of the European Reference Network for Rare Kidney Diseases in collaboration with the European Reference Network for Hereditary Metabolic Disorders and included pediatric and adult nephrologists, metabolic specialists, as well as liver and kidney transplant specialists.

CKD has become a significant clinical issue that requires specific follow-up in both pediatric and adult departments. Creatinine-based formulae significantly overestimate kidney function and the estimation of estimated glomerular filtration rate (eGFR) is more accurate using cystatin C. Besides usual kidney indications, acute dialysis may be required in emergency in case of acute metabolic decompensation to clear metabolic toxins. Long-term dialysis may be initiated for clearance of toxic metabolites. Long hours on hemodialysis (HD) and/or daily dialysis are required. The indications for transplantation in MMA are a high rate of metabolic decompensations, a high burden of disease and difficult metabolic control. Transplantation is also indicated in case of long-term complications. Combined liver-kidney transplantation (LKT) should be preferred in patients with MMA with CKD. Possible calcineurin inhibitors (CNIs) induced neurotoxicity was described in patients with MMA requiring immunosuppressive treatment monitoring and adaptation.

Overall, 13 statements were produced to provide guidance on the management of CKD, dialysis, and transplantation in pediatric and adult patients with MMA.

## Introduction

**Figure 1:**
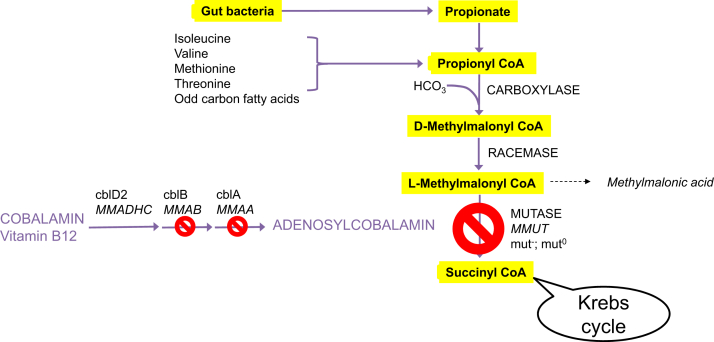
**Methylmalonic acidemia metabolic pathway.** Propionyl-CoA is metabolized in the mitochondria by specific enzymes. Defects in the genes *MMUT, MMAA, MMAB* or to a lesser extent *MMADHC* (more specifically variant 2) lead to isolated MMA. Propionyl-CoA, the precursor of methylmalonyl-CoA is derived from various sources. Accumulating methylmalonyl-CoA in isolated MMA is hydrolyzed to methylmalonic acid, the main biomarker of the disease. Gene names are italicized. CoA, co-enzyme A; MMA, methylmalonic acidemia.

MMAs are a group of rare inherited metabolic diseases affecting propionate catabolism sharing the common feature of elevated concentration of methylmalonic acid in blood, urine, and other body fluids.^1^ This document focuses on classic or isolated MMAs, a group of autosomal recessive disorders whose estimated overall incidence is one in 50,000 newborns.^2^ Isolated MMAs can be caused by pathogenic gene variants in the *MMUT* locus encoding the methylmalonyl-CoA mutase apoenzyme, or by those in genes required for provision of its cofactor, 5’-deoxyadenosylcobalamin (AdoCbl) (Figure 1). Isolated MMA is classified into several genotypic classes and complementation groups (groups of allelic mutations). These are designated either mut^−^ or mut^0^ (together termed mut), according to whether there is minimal or no apoenzyme activity *in vitro*, respectively, or cobalamin A, B or D-variant 2 (cblA/B/D-MMA) for cofactor defects. Approximately one-half to two-thirds of patients with isolated MMA have a mutase apoenzyme defect (mut designation); the remaining patients have cobalamin variants. To date, more than 200 disease-causing pathogenic variants in patients with mut*-*type MMA have been identified at the MMUT locus.^3^

CKD is a common complication of MMA, leading to kidney failure, dialysis, and KT. Occurrence of kidney dysfunction is related to the molecular subtype, mut^0^ patients being affected earlier in life; however, all patients with isolated MMA are at increased risk of developing kidney insufficiency in their long-term course of the disease.^4^ The mechanisms responsible for kidney failure in MMA are not yet fully understood but patients usually present with tubulointerstitial nephropathy, mitochondrial impairment, and alterations that are exacerbated by anomalies in PINK1/Parkin-mediated autophagy with concomitant oxidative and epithelial stress seem to play a key role in the development of this complication.^5^

Moreover, metabolic treatment of the disease may also require dialysis and liver or combined LKT. Because both pediatric and adult nephrologists are directly involved in the management of these patients, in collaboration with metabolic and transplant specialists, the goal of these recommendations was to focus on specific aspects of kidney management, aiming to provide health care professionals working with patients with MMA, clinical practice recommendations for optimal patient care.

## Methods

Development of these clinical practice recommendations is an initiative of the European Reference Network for Rare Kidney Diseases in collaboration with the European Reference Network for Hereditary Metabolic Disorders, spanned from October 2021 until January 2024, and involved 7 meetings, to discuss selected topic areas chosen by the cochairs of the recommendations group. These joint recommendations development group included pediatric and adult nephrologists, metabolic specialists, as well as liver and kidney transplant specialists. Working groups focusing on specific topics were formed.

To ensure that the statements derived from this work could be translated into actionable advice, the core group developed clinical questions based on the elements of the PICO framework: the Patient to whom the recommendation applies, the Intervention under consideration (i.e., treatment or diagnostics), the Comparator (that is, compared with “no action,” placebo or an alternative intervention), and the Outcomes affected by the intervention. A systematic literature review was performed using Medline/PubMed and the Cochrane Library. The following key words were used: “methylmalonic acidemia” OR “methylmalonic acidemia” OR “methylmalonic aciduria” OR “methylmalonic acidemias” OR “methylmalonic acidemias” OR “methylmalonic acidurias” AND “kidney” OR “transplantation” OR “kidney disease” OR “kidney failure” OR “dialysis” OR “peritoneal dialysis” OR “hemodialysis” OR “Continuous kidney replacement therapy.” Articles relevant to the topic of diagnosis and management of MMAs were selected. A total of 299 papers were assessed, and 112 were finally selected for systematic analysis. After an extensive literature review, clinical practice points were developed. Recommendations were graded by the writing committee following the American Academy of Pediatrics recommendations according to their level of agreement after literature review (Supplementary Figure S1).^6^

The statements were then reviewed by an external voting panel of specialists in pediatric and adult nephrology and metabolic medicine and transplant surgeons. The members of the voting panel were asked to provide a level of agreement for all 13 statements on a 5-point scale (strongly disagree, disagree, neither agree nor disagree, agree, strongly agree), according to the Delphi method and to suggest rewording if appropriate.^7^ It was agreed *a priori* that at least a 70% level of consensus was required for each statement, failing which the recommendation would be adapted after discussion in the expert panel, and reviewed again. At least 70% agreement was achieved for 10 out of 13 statements (agree or strongly agree). Of the 3 statements (points 5, 9, and 12) with insufficient agreement, the text was adjusted according to the comments of the voters, discussed within the core group, and again sent to the voting panel. The revised statement was then agreed by >70% members of the voting panel.

## Recommendations

### CKD

#### Incidence of CKD in Patients With MMA and Age at Kidney Disease According to Genotype

With the improved survival of patients with MMA, CKD has become a significant clinical issue. CKD results from chronic tubulointerstitial nephritis, manifesting with a declined kidney function evolving toward kidney failure. Individuals with isolated MMA are thought to be at risk of developing kidney insufficiency. However, the risk of kidney insufficiency is variable and depends on parameters such as disease severity and cobalamin responsiveness. In a series of 30 patients with cobalamin-unresponsive MMA (mut^0^, mut^−^, and cblB), 47% developed CKD.^8^ A larger series, including 82 patients demonstrated that CKD was most common in patients with mut^0^ (61%) and cblB (66%) and occurred less frequently in patients with cblA (21%) and mut^−^ (0%) during the study period.^9^ Median age at CKD presentation was 8 years (mut^0^), 13.5 years (cblB), and 11 years (cblA).^9^ Thus, in terms of kidney impairment, individuals with mut^0^ and cblB-type MMA are considered severely affected regarding morbidity and mortality, whereas mut^−^-type and cblA-type MMA exhibit a more attenuated disease course.^10^ In most patients with MMA who exhibit kidney failure, deterioration of kidney function may occur in a few years, requiring vigilant monitoring.^4^^,^^11^

#### Methods of Assessment of Kidney Function in Patients With MMA

A broad range of functional kidney abnormalities are known to occur in patients with MMA. The main kidney manifestation is a nonspecific chronic tubulointerstitial nephritis.^11^^,^^12^

In contemporary well-managed patient cohorts, isolated potassium loss, or incomplete tubular proximal involvement with elevation of β_2_-microglobulinuria were observed without glycosuria, hematuria, or albuminuria.^11^ Over the years, patients develop progressive CKD, including kidney failure.^4^ Few reports described other signs of tubular dysfunction, such as tubular distal acidosis.^13^

Assessing GFR in patients with MMA may be challenging. The decreased muscle mass seen in patients together with their low protein diet makes routine laboratory markers, such as serum creatinine, poorly predictive of early kidney dysfunction.^4^^,^^13^ Both creatinine-based formulae used in the CKD-Epidemiology Collaboration and Schwartz formulae significantly overestimate kidney function in patients with MMA. Compared to measured GFR, bias estimation of 16 ± 15 ml/min per 1.73 m^2^ and 37 ± 22 ml/min per 1.73 m^2^ have been published for CKD-Epidemiology Collaboration and Schwartz, respectively.^11^ Thus, a creatinine-based formula to calculate eGFR will lead to an underestimation of the true severity of kidney impairment.^11^ The estimation of GFR is more accurate using cystatin C instead of creatinine, because it is independent from muscle mass.^4^^,^^14^ Measured GFR using iohexol, inulin, or Cr-EDTA clearance can be performed when available.^4^^,^^11^ Kidney biopsy is generally not required. The frequency of measurement is based on the patient’s age, CKD stage, and severity of metabolic disease (Table 1).

**Table 1:** Clinical and laboratory parameters follow-up in patients with MMA (in stable situations)

Assessment
NH3, lactate
Quantitative plasma amino acids (3-4 hours of fasting prior to sample collection)
MMA in plasma and urine if available depending on local experience and kidney function
Acylcarnitine profile in dried blood or plasma
Diet history
Growth (weight, length or height, head circumference)
Full clinical examination
Albumin, prealbumin, total protein
Bone health (Ca, P, ALP, Mg, PTH, 25-OH vitamin D in blood; Ca, P in urine)
Full blood count, ferritin, folic acid, vitamin B12
Neurological examination with assessment of developmental milestones
Kidney function (serum creatinine, eGFR, electrolytes, cystatin C, urinary electrolytes and protein loss) Iohexol GFR (when available, in case of important clinical decisions)
Pancreas function (lipase, pancreatic amylase)
Cardiac assessment (ECG, echocardiography)
Formal developmental/cognitive assessment
Cerebral MRI (EEG, EMG if clinically indicated)
Ophthalmologic assessment
Formal hearing test

ECG, electrocardiogram; EEG, electroencephalogram; eGFR, estimated glomerular filtration rate; EMG, electromyography; MMA, methylmalonic acidemia; MRI, magnetic resonance imaging.

The rate of kidney growth in MMA individuals is impaired by about one-third to one-half of normal values.^14^ Kidney growth is predicted by height^13^ and correlates negatively with serum cystatin C and plasma MMA concentrations (pMMA).^4^ In order to evaluate kidney growth in children, repeated kidney ultrasound could be proposed.

Proximal tubular function may be assessed by measurement of plasma electrolytes, uric acid, acid base balance, low molecular weight proteinuria, calcium phosphate balance. Less commonly, other signs of proximal tubular dysfunction have been described (such as proximal tubular acidosis and kidney glycosuria).

**Clinical Practice Point 1**

• Because both CKD-Epidemiology Collaboration and Schwartz formulae significantly overestimate renal function in patients with MMA, use cystatin C based equations rather than creatinine-based equations for the estimation of GFR. **Grade B, strong.**
• Measured GFR using iohexol clearance can be performed when possible. **Grade B, moderate.**

#### Specific Aspects of CKD Management in Patients With MMA: Monitoring Clinical and Laboratory Parameters

Currently in some countries, patients with MMA come to medical attention through newborn screening, enabling early initiation of treatment.^15^ However, despite early and appropriate therapeutic management, a relevant proportion of patients may still become symptomatic before the first screening results are available and are still prone to develop long-term complications, including kidney failure. Given that there is no evidence for specific treatment strategies that preserve kidney function in patients with MMA, following standard protocols for CKD is suggested.^4^ Given the high risk of pancreatitis in children with MMA, and that pancreatitis may be triggered by hypercalcemia, careful monitoring of serum calcium and vitamin D levels, and adjusting medications to keep serum calcium in the low-normal range is recommended.

**Clinical Practice Point 2**

• Given that there is no evidence for specific treatment strategies that preserve kidney function in patients with MMA, we suggest following standard recommendations for CKD impairment. **Grade C, moderate.**

Standard long-term management of patients with MMA includes^2^:

1. Life-long low protein diet and increased caloric intake, to reduce protein catabolism and MMA accumulation; adapted to patient needs over time.^16^
2. Administration of oral L-carnitine (100–200 mg/kg/d in 2–4 doses), aiming at buffering and eliminating the excess of propionyl-CoA, the precursor of methylmalonyl-CoA, which is supposed to be responsible for some of the toxic metabolite effects in MMA. Carnitine also restores CoA levels and replenishes intracellular carnitine stores (4).
3. Vitamin B_12_ (hydroxocobalamin) in cobalamin-responsive patients with MMA. Note that cobalamin responsiveness should be tested in a standardized way.^4^
4. Acute and long-term treatment with carglumic acid (N-carbamylglutamate), a structural analog of N-acetyl-glutamate which stimulates carbamoylphosphate synthase and promotes the removal of ammonia via the urea cycle, may be beneficial for the management of hyperammonemia.^17^
5. Consider rhGH treatment in selected cases with CKD.
6. Bicarbonate supplementation according to acid base balance.

Kidney function needs to be monitored on a regular basis as described above.^4^ Potential other kidney injury factors should be minimized and complications of CKD monitored as in general population, as follows:

1. monitoring electrolyte and acid base balance
2. regular screening for hypertension and albuminuria
3. anemia
4. secondary hyperparathyroidism and renal osteodystrophy treated with vitamin D.^18^^,^^19^

Nephrologists should be aware of the need for optimal control of the metabolic disorder assessed by plasma ammonia and lactate concentrations, and acid balance status. Monitoring diet and metabolic profile (pMMA and urinary MMA concentration, plasma amino acids, including glutamine and glycine) helps to identify those patients with increased risk of progression to kidney failure.^20^ pMMA excretion is reduced when GFR reduces, leading to an accumulation of pMMA in the body that, in turn, causes a further deterioration in kidney function and a vicious cycle of worsening metabolic and kidney function.^11^ Thus, pMMA monitoring rather than urine MMA may be more accurate as kidney function decreases and in dialysis patients (Table 1).

Height needs to be assessed regularly. Impaired growth is a common manifestation of CKD in children. Furthermore, patients with MMA often experience failure to thrive and poor growth, mainly in patients with more severe disease subtype (mut^0^). Impaired growth has been associated with the underlying disease and natural protein-restriction, mainly to overly restricted or unbalanced diet.^4^ Thus, decreased growth may indicate both kidney involvement and suboptimal management of MMA. Underlying mitochondrial dysfunction could possibly play a role in reduced patient growth.^10^

#### Adaptation of Metabolic Treatment and Diet to CKD Stage

The progression of kidney disease impacts metabolic balance and nutritional intervention: consider dialysis and transplantation or preemptive transplantation as kidney function decreases.

Metabolic follow-up is mainly based on pMMA concentration because urinary MMA excretion is no longer a good biomarker in individuals with CKD. However, it should be considered that pMMA concentration increases with patient’s age in relation to the progression of CKD, because it negatively correlates with residual kidney function.^21^^,^^22^ Optimal dietary management should aim at preventing protein malnutrition subsequently aggravating protein catabolism and hence chronic intoxication through enhanced pMMA accumulation.^4^ Expert dietetic and nutritional support is highly recommended. Metabolic follow-up also includes other parameters such as ammonia, lactate, amino acid levels, and acid base status. Adequate hydration based on urine output and CKD stage is recommended.

**Clinical Practice Point 3**

• In patients with MMA with CKD, the metabolic follow-up should rely on pMMA concentration rather than urinary MMA concentration and should include other parameters such as ammonia, lactate, amino acid levels, and acid base status. Expert dietetic support should ensure adequate nutritional status. **Grade B, strong.**

### Dialysis

#### Indications for Starting Dialysis

Patients with MMA may require short-term dialysis for an acute metabolic decompensation or acute kidney injury, or even long-term (or maintenance) dialysis.^2^^,^^4^

Metabolic decompensation is a life-threatening emergency and clearance of toxic metabolites toxins must be performed as soon as possible. Acute dialysis is required in the following acute situations when initial optimal medical management fails acute severe neurological symptoms, change in level of consciousness, persistent ketoacidosis with rapidly increasing or high plasma ammonium and/or lactate level. It is important to note that clinicians should not wait for results of plasma and/or urine MMA levels, which are usually not available in an emergency, prior to starting dialysis. Treatment must be individualized and adjusted per patient and clinical scenario.

Long-term dialysis may be required for clearance of toxic metabolites; therefore, dialysis is often initiated at higher eGFR levels compared to those without MMA. Because eGFR is not a reliable measure, it should not be the major criteria to determine when dialysis is required in patients with MMA.^11^

The parameters that indicate the need for maintenance dialysis therapy are:

1. Difficulties to maintain metabolic stability, including increasing high pMMA levels, despite optimized controlled protein intake and medications.
2. Preventing signs and symptoms of protein malnutrition (muscle and weight loss, thinning hair, lighter hair, hair loss), because of impossibility to increase protein intake without a risk of chronic high pMMA concentrations and subsequent neurological acute episodes. One of the risks of malnutrition is sarcopenia with further healing impairments and complications after the dialysis period, during the acute phase of transplantation.
3. Very rarely, severe recurrent pancreatitis not controlled by conventional treatment.

The decision to start maintenance dialysis is however based on an individualized approach.

**Clinical Practice Point 4**

• Acute metabolic decompensation is a life-threatening emergency. Clearance of toxic metabolites and correction of clinical and laboratory parameters by dialysis must be performed as soon as possible when initial optimal medical management failed. **Grade B, strong.**
• Long-term dialysis may be required for clearance of toxic metabolites. Start dialysis when pMMA concentrations are rising with metabolic or clinical instability despite an optimized controlled protein intake and medications. Dialysis may be required at higher eGFR levels compared to those without MMA. **Grade C, moderate.**
• Do not decrease protein intake to control pMMA concentrations. **Grade C, moderate.**

#### Preferred Dialysis Modality for Treating Children and Adults With MMA

Acute MMA decompensation requiring dialysis is a life-threatening emergency and must be treated urgently in any center where dialysis can be safely performed. After stabilization, the patient should be transferred to a center that has multiprofessional expertise in treating metabolic and kidney disorders. The preferred dialysis modality will depend on the availability and expertise in each center, particularly in the case of dialysis for infants.^23^^,^^24^ However, continuous veno-venous HD or intermittent HD are preferred to peritoneal dialysis (PD) to achieve rapid metabolic control.^2^^,^^4^

pMMA is a low molecular weight substance (molecular weight 118.09 Daltons) and is therefore easily cleared by diffusion on PD or HD. However, pMMA is constantly produced by the body; thus, the rate of pMMA generation exceeds the capacity of intermittent dialysis therapies such as PD or HD to clear it, and pMMA level rebounds despite efficient dialytic clearance. In the case of chronic dialysis, there is currently no literature to suggest that any of these techniques is superior to others. There are only a few case reports with chronic PD; and recommendations are based on expert opinion.^4^^,^^25^^,^^26^ PD seems to have a good effectiveness on pMMA clearance, especially in infants, probably because of the large size of their peritoneum proportional to body weight. HD may be more effective in adults. The choice of dialysis modality must be selected in discussion with the patients (where appropriate) and their caregivers.^27^

Therefore, frequent daily or long hours on HD or daily PD are necessary for effective pMMA clearance. HD sessions should last 4 or 5 hours per session with at least 4 sessions per week. The number of sessions should not be decreased because of pMMA level rebound and adapted to predialysis pMMA levels.^28^ Bicarbonate-based dialysis fluid must be used for PD in preference to lactate-based dialysis fluids whenever possible, and avoid acetate based HD fluids.^25^

It may be noted that intensive dialysis regimens that combine diffusive and convective mechanisms such as hemodiafiltration will not achieve better pMMA clearance because pMMA is cleared entirely by diffusion. In addition, there is no added benefit to combining PD and HD simultaneously to improve pMMA clearances; this greatly increases the risk of infections and increases the burden of care. Instead, dialysis may be considered as a bridge to transplantation.

**Clinical Practice Point 5**

• **Acute Dialysis.** Continuous veno-venous HD or intermittent HD are preferred to PD to achieve rapid metabolic control. **Grade C, moderate.**
• If there are technical difficulties with performing HD, PD may be considered until the infant or child is moved to a center with appropriate dialysis facilities. **Grade C, weak.**
• **Long-Term (or Chronic) Dialysis.** HD and PD are both effective dialysis modalities in the long-term treatment of patients with MMA. HD may be more effective; however, PD represents a good option in infants. **Grade C, moderate.**
• Long hours on HD or frequent daily dialysis are required to achieve optimal pMMA clearance. **Grade C, moderate.**
• Use bicarbonate-based dialysis fluid in preference to acetate or lactate-based dialysis fluids for HD and PD**. Grade X, strong.**

#### Adaptation of Metabolic Treatment and Diet in Dialysis Patients

In this part, only specific aspects of dialysis in patients with MMA are described. All the general clinical practices are described in the Kidney Disease Improving Global Outcomes guidelines and International Society for Peritoneal Dialysis guidelines.^29^^,^^30^ Apart from the usual treatment and diet recommended in dialysis, there are some specificities in case of MMA. The following recommendations are based on expert opinion. On dialysis, the total protein intake can be maximized (according to biochemical profile) to prevent chronic protein malnutrition, while the patient is waiting for transplantation.

In case of PD, attention should be paid to:

1. Loss of protein in dialysis fluid, which should be considered for the adaptation of protein intake.
2. Additional calories coming from glucose-based PD dialysis fluid.
3. Loss of sodium and potassium in dialysate, and a need to increase sodium and potassium supplementation.
4. Loss of water-soluble vitamins in dialysis fluid, and a need to increase supplementation.

**Clinical Practice Point 6**

• On dialysis, the natural protein intake can be increased (according to biochemical profile) to prevent chronic protein malnutrition. **Grade C, moderate.**

#### Monitoring of Clinical and Laboratory Parameters

Apart from the usual parameters monitored in dialysis and parameters already described in the CKD section, there are some specificities in case of patients with MMA on dialysis.^29^^,^^31^ The next recommendations are based on expert opinion.

##### HD

Monitoring pMMA concentrations regularly before and after dialysis session is useful to adapt the duration and modalities of dialysis sessions. Samples should be analyzed at the start of the session to analyze the general trend of baseline level and at the end of the session to measure its effectiveness.

Free carnitine plasma concentration should be monitored because lifelong carnitine supplementation is recommended in patients with MMA, but losses may occur in dialysis patients.^32^ Carnitine supplementation should therefore be tailored to the losses due to HD. Plasma amino acid measurements allow to assess essential amino acid concentrations and to adapt protein intake.

##### PD

Monitoring pMMA and amino acid concentrations is useful to adapt dialysis prescription and protein intake. Due to muscle wasting (sarcopenia) in patients with MMA, the risk of hernia is increased and should be monitored.

### Transplantation

#### Indications for Transplantation in Patients With MMA

Liver or combined LKT should be considered in all patients with severe disease course regardless of the genotype. However, patients with mut^0^ genotype usually exhibit a more severe outcome, including kidney impairment. The indications for organ transplantation in MMA are a high rate of metabolic decompensations, high burden of disease and difficult metabolic control with consequent high risk of long-term complications, such as CKD, neurological deterioration, chronic pancreatitis, and/or optic neuropathy.^33^, ^34^, ^35^, ^36^, ^37^, ^38^ Although organ transplantation has a positive impact on the long-term clinical outcomes, it is not curative. There are reported cases of acute neurological events and/or progression of neurological disease after liver or LKT.^36^^,^^39^

**Clinical Practice Point 7**

• Liver or combined LKT should be considered in all patients with severe disease course regardless of the genotype. The indications for transplantation in MMA are a high rate of metabolic decompensations, a high burden of disease and difficult metabolic control. **Grade C, moderate.**

#### When Should We Decide to Perform Transplantation?

The scenery of transplantation in MMA shows 2 types of indications. Transplantation should be first considered in young patients with poor metabolic control and frequent hospitalizations, having a high risk of acute neurological damage, high risk of death, and a poor quality of life. The other indications for transplantation are the long-term complications in particular CKD and to minimize the risk of further neurological complications.

The increasing implementation of expanded newborn screening programs, which may include MMA in the disease panel, can lead to early identification of young patients with severe genotype in whom treatment can be offered early, ideally at a presymptomatic stage, and in whom isolated liver transplantation could be considered.^40^

To ensure good clinical outcomes and reduce mortality and complications related to the surgical procedure, it is ideally advisable that the patient has achieved a metabolic stability at the time of transplantation and that the intervention is carried out by an experienced multidisciplinary transplantation team including surgeon, anesthesiologist, metabolic specialist, nephrologist, cardiologist, dietician, and psychologist.^33^^,^^35^^,^^40^, ^41^, ^42^

**Clinical Practice Point 8**

• Transplantation should be considered in young patients with poor metabolic control and frequent hospitalizations. Transplantation is also indicated in case of long-term complications, in particular CKD and to minimize the risk of further neurological complications. **Grade C, moderate.**

#### The Choice of Transplantation: Kidney, Liver or Combined Liver-Kidney?

The case of each patient should be discussed individually. Liver transplantation should be chosen in patients with MMA with normal kidney function or mild CKD (preferably based on eGFR by cystatin-based formula or, if available, measured GFR), whereas combined LKT should be preferred in patients with MMA with CKD stage 3b, 4, and 5. The KT alone allows to correct kidney function but does not reduce the pMMA concentrations as much as LKT. This is because the kidney contains a lower amount of functioning enzyme compared to the liver.^34^^,^^43^^,^^44^ In the setting of isolated liver transplantation patients, KT may be performed later if kidney function starts to deteriorate and leads to kidney failure. Isolated KT may be discussed situations, including adults (especially cblA patients) and late-onset forms because it is associated with less morbidity or children with limited life expectancy.

From a surgical point of view, smaller patients (presymptomatic scenario) have a greater chance of getting liver transplantation than larger patients (25–30 kg vs. heavier weight) due to the possibility of using partial grafts from split liver and living donor. Patients with MMA can be transplanted with partial liver grafts, including those coming from living donors, without increased metabolic risks compared to whole liver transplantation.

**Clinical Practice Point 9**

• Liver transplantation should be considered early and before CKD progresses to improve metabolic control, reduce neurological risk, and minimize late multi-organ complications and disease burden. Combined LKT should be preferred in patients with MMA with CKD stage 3b, 4, and 5. **Grade C, moderate.**

#### Specific Procedures Before and During Surgery of Transplantation

Before transplantation, a cardiac assessment should be performed, including blood pressure measurement, electrocardiogram, and echocardiography, as in other transplant candidates. Cardiac magnetic resonance imaging might be considered for patients with any echography anomaly. A neurological evaluation is required, including a developmental evaluation and brain magnetic resonance imaging. An ophthalmologic examination is recommended.

The perioperative treatment aims to prevent catabolism and avoid metabolic decompensation.^29^ Fasting should be maintained according to the minimal anesthetic requirements and replaced by balanced amino acid free glucose (with electrolytes) infusions containing 10% glucose and lipids at the appropriate age-dependent calories intake to block lipolysis. Continuous i.v. L-carnitine should be started. The use of low-dose insulin can be considered due to its anabolic effects if needed to treat recalcitrant hyperglycemia.^2^ There is no data on the potential benefits for perioperative HD in patients with MMA.^28^ In case of CKD, HD could be considered before surgery. Continuous veno-venous HD should be available during the procedure for metabolic control according to lactate, ammonia, and metabolic acidosis. To reduce the risk of metabolic decompensation during transplantation, strategies should be adopted which minimize the times of caval and portal clamping.

Immediately after transplant, lactate should be checked often (every 4 hours) because metabolic acidosis may occur. In case of a high lactate level, dialysis could be started. Vitamin B1 (thiamine) should also be administered in a pharmacological dose in this acute context.

Parenteral nutrition might be required immediately posttransplant; however, the goal is to transition to enteral feeding as early as possible. Parenteral proteins should be introduced gradually as soon as possible based on biochemical parameters.

Glucose infusion (from 4 mg/kg/min to 8 mg/kg/min according to age) in the perioperative management requires close monitoring due to the risk of hyperlactatemia and/or hyperglycemia.

Electrolytes including phosphate should be monitored.

**Clinical Practice Point 10**

• During surgery, fasting should be prevented by balanced glucose infusions containing 10% glucose with electrolytes (and lipids) at the appropriate age-dependent calories intake to block catabolism during procedure. i.v., L-carnitine should be added. **Grade B, strong.**
• In the case of CKD, HD could be considered before surgery. **Grade C, moderate**.

#### Expected Benefits of Transplantation

Overall, benefits include improved clinical outcomes and reduction in pMMA levels leading to less of an effect on organs. Apart from treatment of kidney failure for LKT (and KT), LKT and liver transplantation have been proven to significantly reduce pMMA concentration, metabolic decompensations, pancreatitis, and hospitalization rates in several cohorts of patients.^34^^,^^40^^,^^41^^,^^45^, ^46^, ^47^ Neurological progress has been reported.^33^^,^^34^^,^^37^^,^^41^^,^^47^ Improvement of weight and height after liver transplantation in young patients has been documented.^48^ For these reasons, the quality of life of patients and their families could be improved, as so far demonstrated only in a few studies.^49^

#### Adaptation of the Metabolic Treatment and Diet After Transplantation

After transplantation, patients with MMA should be continuously supplemented with L-carnitine due to persistent extrahepatic production of toxic propionyl-CoA.^33^, ^34^, ^35^^,^^37^^,^^46^

The optimal diet composition after transplantation is still debated. There is no agreement on the amount of protein intake after liver transplantation or LKT; some authors report a progressive natural protein intake increase in childhood,^34^, ^35^, ^36^ others report conservation of protein restriction.^28^^,^^46^ In our experience, patients can tolerate a natural protein intake of approximately 0.8 g/kg in adults, up to 1.5 g/kg in children, according to CKD requirements, discussed case by case and adjusted according to the monitoring of the recommended biomarkers^4^: lactate, plasma and urinary MMA, and plasma aminoacids.^34^^,^^45^ Exploratory measurements may include plasma methylcitric acid and FGF21.^21^

A frequent withdrawal of enteral nutrition after transplantation is observed due to less frequent nausea^33^^,^^34^^,^^40^^,^^50^ and almost all patients are fed by mouth within 12 months after transplantation.

**Clinical Practice Point 11**

• After transplantation, patients with MMA should continue supplementation with L-carnitine. **Grade B, strong**.
• After liver or combined LKT, it is possible to progressively increase natural protein intake (as compared to pretransplantation), adjusted according to the biochemical profile and individual tolerance. **Grade C, moderate.**

#### Preferred Immunosuppressive Regimen

The choice of immunosuppression is based on age (adult vs. pediatric) and the type of transplant (liver vs. kidney vs. liver and kidney). Posttransplant immunosuppression is generally based on induction agent as per the local protocols and CNIs (tacrolimus and cyclosporine), and/or mycophenolate and/or steroids. Possible CNI-induced neurotoxicity has been described in patients with MMA that can present with a variety of symptoms such as confusion, tremor, seizures, cerebral hemorrhage, ischemic stroke, stroke or stroke-like episodes, Leigh-like lesions, and posterior reversible encephalopathy syndrome.^33^^,^^34^^,^^51^, ^52^, ^53^ These symptoms can also be mistaken for characteristic neurological findings in patients with MMA.

Tacrolimus, the CNI usually used in solid organ transplantation, may be associated with sudden onset of tremor (basal ganglia toxicity) in all patients and is particularly poorly tolerated in patients with MMA. Given that CNI-induced neurotoxicity may be clinically indistinguishable from a metabolic stroke, a careful clinical, biochemical, and neuroradiological assessment is required to distinguish between both entities whose therapy is strikingly different, with prompt tacrolimus reduction or discontinuation in CNI-induced neurotoxicity.

In the early course after transplantation, we recommend the use of CNI with an increasing evidence that cyclosporine has a more favorable tolerance profile than tacrolimus.^54^, ^55^, ^56^ A thorough pharmacokinetic monitoring is recommended, associated with a strict control of the other parameters associated with neurotoxicity (triglycerides, cholesterol, and electrolytes), even if neurotoxicity is not directly dependent on plasma levels.

For maintenance therapy, mTOR inhibitors could be considered alternatively to avoid CNI in adults.^34^^,^^51^

**Clinical Practice Point 12**

• In the early course after transplantation, we recommend the use of CNI with an increasing evidence that cyclosporine has a more favorable tolerance profile than tacrolimus.
• For maintenance therapy, mTOR inhibitors could be considered alternatively to avoid CNI in adults. **Grade C, weak.**

#### Potential Complications of Transplantation

Patients affected by MMA are highly complex and carry a high lifelong health burden. Older series report increased risks of mortality and morbidity, whereas more recent ones report better short-term and medium-term results, in children.^33^^,^^37^ A multidisciplinary approach is recommended.

Although rare, there is a persistent risk of metabolic decompensation after transplantation.^39^ Neurological complications are described above.^33^^,^^34^^,^^51^ Other complications are like other transplant patients. In combined LKT, there are more surgical vascular complications (14%) and higher risk of rejection (22.2%) than after liver transplantation (3% and 13.4% respectively).^41^

#### Clinical, Laboratory Parameters and Other Instrumental Parameters Monitoring

It is mandatory to continue a metabolic follow-up after transplantation. A multidisciplinary team should be involved and include specialists in metabolism, organ transplant, nephrology, hepatology and diet.

Patients should be followed-up with as per transplant protocol. Clinical and biochemical parameters should be monitored after transplantation, particularly anthropometric indices; kidney function biomarkers (creatinine, GFR, and cystatin C); and metabolic biomarkers such as blood lactate and pMMA^34^^,^^46^^,^^47^ (Table 1). FGF21 seems to be an interesting biomarker for the occurrence of long-term mitochondrial complications. After transplantation, a decrease in FGF21 levels has been observed. A significant reduction of FGF21 has been observed after liver transplantation and LKT; however, levels remain markedly elevated after solely KT.^21^

During follow-up after transplantation, neurological monitoring is recommended and may include serial neuroradiological study (magnetic resonance imaging), neurophysiological examination (electroencephalogram, brainstem-evoked potential) and neuropsychological test, in case of acute event.^52^ Visual function should also be assessed in the long-term follow-up (functional evaluation, fundoscopy).

MMA has a substantial impact on health-related quality of life (HrQoL). Free comments suggested a positive impact of liver transplantation on general health, family social involvement, parental anxiety, outlook, and on intellectual and emotional health of the child. One publication analyzed HrQoL after liver transplantation and did not find significant differences in liver transplantation and non-liver transplantation patient scores on the PedsQL scales.^49^ In another series, improvement of HrQoL after liver transplantation was described based on unscheduled admission days, tube feeding, and anxiety.^50^ A more recent study evaluated the impact of liver transplantation on HrQoL in a cohort of patients with intoxication type metabolic diseases, including 6 with MMA, by using 2 different tools, the “generic” PedsQL; and MetabQoL, a specifically designed tool for this disease category.^57^ The study demonstrated a post-transplant improvement of HrQol in all diseases, suggesting a higher sensitivity of MetabQoL in the assessment of disease-specific domains.^57^

**Clinical Practice Point 13**

• After transplantation, a multidisciplinary team should continue to be involved and include metabolic and transplant specialists, nephrologist, hepatologist, cardiologist and dieticians. **Grade B, strong.**

## Conclusion

Overall, 13 statements were produced to provide guidance on management of CKD, dialysis and transplantation in patients with MMA from both the kidney and metabolic points of view (Table 2). Briefly, CKD represents a significant clinical issue in MMA but requires a very specific follow-up in both pediatric and adult departments. Creatinine-based formulae significantly overestimate kidney function in patients with MMA and the estimation of eGFR is more accurate using cystatin C. As kidney function decreases, metabolic follow-up should be based on pMMA concentration because urinary MMA excretion is no longer reliable. Besides usual kidney indications, acute dialysis may be required in emergency in case of acute metabolic decompensation to clear toxic metabolites. Long-term dialysis may be initiated for clearance of metabolic toxins.

**Table 2:** Summary of recommendations

	Clinical practice point	Evidence
1	1. As both CKD-EPI and Schwartz formulae significantly overestimate renal function in patients with MMA, use cystatin C based equations rather than creatinine-based equations for the estimation of GFR.	B, strong
	2. Measured GFR using iohexol clearance can be performed when possible.	B, moderate
2	Since, there is no evidence for specific treatment strategies that preserve kidney function in patients with MMA, we suggest following standard recommendations for CKD impairment.	C, moderate
3	In patients with MMA with CKD, the metabolic follow-up should rely on pMMA concentration rather than urinary MMA concentration and should also include other parameters such as ammonia, lactate, amino acid levels, and acid base status. Expert dietetic support should ensure adequate nutritional status.	B, strong
4	1. Acute metabolic decompensation is a life-threatening emergency. Clearance of toxic metabolites and correction of clinical and laboratory parameters by dialysis must be performed as soon as possible when initial optimal medical management fails.	B, strong
	2. Long-term dialysis may be required for clearance of toxic metabolites.	C, moderate
	- Start dialysis when pMMA concentrations are rising with metabolic or clinical instability despite an optimized controlled protein intake and medications.	
	- Dialysis may be required at higher eGFR levels compared to those without MMA.	
	3. Do not decrease protein intake in an attempt to control pMMA concentrations.	C, moderate
5	1. Acute dialysis:	
	- Continuous veno-venous HD or intermittent HD are preferred to PD to achieve rapid metabolic control	C, moderate
	- If there are technical difficulties with performing HD, PD may be considered until the infant or child is moved to a center with appropriate dialysis facilities.	C, weak
	2. Long-term (or chronic) dialysis:	
	- HD and PD are both effective dialysis modalities in the long-term treatment of patients with MMA. HD may be more effective, but PD represents a good option in infants.	C, moderate
	- Long hours on HD or frequent daily dialysis are required to achieve optimal MMA clearance.	C, moderate
	- Use bicarbonate-based dialysis fluid in preference to acetate or lactate-based dialysis fluids for HD and PD.	X, strong
6	On dialysis, the natural protein intake can be increased (according to biochemical profile) to prevent chronic protein malnutrition.	C, moderate
7	Liver or combined liver-kidney transplantation should be considered in all patients with severe disease course regardless of the genotype.	C, moderate
	The indications for transplantation in MMA are a high rate of metabolic decompensations, a high burden of disease and difficult metabolic control.	
8	Transplantation should be considered in young patients with poor metabolic control and frequent hospitalizations.	C, moderate
	Transplantation is also indicated in case of long-term complications, in particular CKD and to minimize the risk of further neurological complications.	
9	Liver transplantation should be considered early and before CKD progresses to improve metabolic control, reduce neurological risk, minimize late multi-organ complications and disease burden.	C, moderate
	Combined liver-kidney transplantation should be preferred in patients with MMA with CKD stage 3b, 4, and 5.	
10	During surgery, fasting should be prevented by balanced glucose infusions containing 10% glucose with electrolytes (and lipids) at the appropriate age-dependent calories intake to block catabolism during procedure. i.v. L-carnitine should be added.	B, strong
	In case of CKD, HD could be considered before surgery.	C, moderate
11	After transplantation, patients with MMA should continue supplementation with L-carnitine.	B, strong
	After liver or combined liver-kidney transplantation, it is possible to progressively increase natural protein intake (as compared to pre-transplantation), adjusted according to the biochemical profile and individual tolerance.	C, moderate
12	In the early course after transplantation, we recommend the use of CNI, with an increasing evidence that cyclosporine has a more favorable tolerance profile than tacrolimus.	C, weak
	For maintenance therapy, mTOR inhibitors could be considered alternatively to avoid CNI in adults.	
13	After transplantation, a multidisciplinary team should continue to be involved and include, metabolic and transplant specialists, nephrologist, hepatologist, cardiologist and dieticians.	B, strong

CKD, chronic kidney disease; CKD-EPI, CKD-Epidemiology Collaboration; CNI, calcineurin inhibitors; HD, hemodialysis; MMA, methylmalonic acidemia; PD, peritoneal dialysis; pMMA, plasma MMA.

Indications for dialysis start are metabolic instability despite an optimized controlled protein intake and medications with elevated pMMA levels. In acute situations, continuous veno-venous HD or intermittent HD are preferred to PD to achieve rapid metabolic control. HD and PD are both effective in the long-term treatment of patients with MMA. Long hours on HD and/or frequent daily dialysis are required to achieve optimal pMMA clearance. On dialysis, the total protein intake can be maximized to prevent chronic protein malnutrition and optimize transplantation. The indications for transplantation in MMA are a high rate of metabolic decompensations, a high burden of disease and difficult metabolic control. Transplantation is also indicated in case of long-term complications, in particular CKD and neurological complications. Combined LKT should be preferred in patients with MMA with CKD. After transplantation, patients will tolerate an increased natural protein intake. Due to its neurotoxicity in patients with MMA, we suggest using immunosuppressive regimen without tacrolimus. After transplantation, a multidisciplinary team should be involved in follow-up of these patients.

Several general guidelines for MMA management have already been published.^2^^,^^4^ Points emphasized in the present paper include a comprehensive and practical description of specific management of CKD, dialysis, and transplantation in patients with MMA elaborated by a multidisciplinary team with clinical experience in the management of these patients, based on recent data. Adult nephrologists and pediatricians should be aware of the specificities of these patients as outcomes have improved. An increasing number of patients now reach adulthood and require specialized care. However, there are still significant knowledge gaps regarding the optimal management of patients with MMA and better-quality evidence is required. We believe that these recommendations will have a positive impact on the outcomes of patients by establishing common standards and spreading and harmonizing good practices.

## Disclosure

MSc reports consulting fees from LogicBio. CDV reports consulting fees from Mamoxi, lectures fees from Nutricia and Piam Farmaceutici, travel support for attending meeting from Piam Farmaceutici, and advisory board fees from Moderna and Immedica. AB report support for attending meeting from Sanofi. Apart from the authors listed, the other authors declared no competing interests.

## Supplementary Material

Supplementary File (PDF) **Supplementary Figure S1.** Matrix for grading of evidence and assigning strength of recommendations according to the American Academy of Pediatrics.^6^

## Footnotes

Supplementary File (PDF)
**Supplementary Figure S1.** Matrix for grading of evidence and assigning strength of recommendations according to the American Academy of Pediatrics.^6^
