# Supplementary material for: Clinical Practice Recommendations on Kidney Management in Methylmalonic Acidemia: an Expert Consensus Statement From ERKNet and MetabERN
Source: Kidney Int Rep. 2024 Sep 6;9(12):3362–74. doi: 10.1016/j.ekir.2024.09.002 (PMC11652068; doi:10.1016/j.ekir.2024.09.002)
Supplement: Supplementary File (PDF) — Supplementary Figure S1. Matrix for grading of evidence and assigning strength of recommendations according to the American Academy of Pediatrics.6 [file mmc1.pdf]

Aggregate evidence quality	Benefit or harm predominates	Benefit and harm balanced
**Level A**<br>• Intervention: well-designed and conducted trials, meta-analyses on applicable populations<br>• Diagnosis: independent gold-standard studies of applicable populations	Strong recommendation	Weak recommendation (based on balance of benefit and harm)
**Level B**<br>Trials or diagnostic studies with minor limitations; consistent findings from multiple observational studies	Moderate recommendation	
**Level C**<br>Single or few observational studies or multiple studies with inconsistent findings or major limitations		
**Level D**<br>Expert opinion, case reports, reasoning from first principles	Weak recommendation (based on low-quality evidence)	No recommendation may be made
**Level X**<br>Exceptional situations where validating studies cannot be performed and benefit or harm clearly predominates	Strong recommendation<br>Moderate recommendation	
